# Supplementary material for: Transport of N-CD and Pre-Sorbed Pb in Saturated Porous Media
Source: Molecules. 2020 Nov 25;25(23):5518. doi: 10.3390/molecules25235518 (PMC7728100; doi:10.3390/molecules25235518)
Supplement: Supplementary file 1 [file molecules-25-05518-s001.pdf]

Transport of N-CD and pre-sorbed Pb in saturated porous media

Salahaddin Kamrani[a,b,]*, Vahab Amiri[c], Mosleh Kamrani[d], Mohammed Baalousha[e,f,]*

[a] Energy Technologies Development Headquarter, PDST, Tehran, Iran
[b] Department of Applied Geology, Faculty of Earth Sciences, Kharazmi University, Tehran, Iran
[c] Department of Geology, Faculty of Science, Yazd University, Yazd, Iran
[d] Department of Chemical Engineering, Faculty of Engineering, University of Kurdistan, Sanandaj, Iran
[e] Center for Environmental Nanoscience and Risk, Arnold School of Public Health, University of South Carolina, USA
[f] Department of Environmental Health Sciences, Arnold School of Public Health, University of South Carolina, USA

N-CDs and N-CD-Quartz surface interaction energy

Based on the DLVO theory, the total interaction potential between particles is the sum of Lisfshitz-van der Waals (ϕ_{VDW}) and electric double layer interactions (ϕ_{EDL}) for N-CDs interacting with quartz. The total interaction potential can be determined using the following expression (Jiang et al 2012; Fan et al 2015).

The ϕ_{VDW} and ϕ_{EDL} can be determined using the following expression (Gregory 1981; Lanphere et al 2013; Trefalt et al 2014):

$$\phi_{VDW} = -\frac{Ar_1r_2}{6h(r_1+r_2)}(1+\frac{14h}{\lambda})^{-1} \quad \text{Eq.1}$$

$$\phi_{EDL}(h) = \pi r\varepsilon_r\varepsilon_0\{2\psi_1\psi_2 \ln\left[\frac{1+\exp(-Kh)}{1-\exp(-Kh)}\right] + (\psi_1^2+\psi_2^2)\ln[1-\exp(-2Kh)]\} \quad \text{Eq.2}$$

In Eq.1, A is the Hamaker constant (1.12×10^{-20} J), r_1 and r_2 are the radii of sphere 1 (N-CD) and 2 (N-CD/quartz), respectively, h is the separation distance between sphere 1 and 2, λ is the characteristic wavelength.

In Eq.2, ε_o is the permittivity of free space, ε_r is the relative permittivity of water, $\psi 1$ and $\psi 2$ are the surface potentials of the N-CD and quartz grain, respectively. Zeta potential

values were used in place of surface potential values for ϕ_{EDL} calculations (Bradford et al 2007; Feriancikova and Xu, 2012) and κ is the Debye reciprocal length which can be calculated using Eq. 3 (Bradford and Torkzaban 2008):

$$K = \sqrt{\frac{e^2 \sum_i n_{j0} Z_e^2}{\varepsilon_r \varepsilon_0 k_b T_k}} \qquad \text{Eq. 3}$$

Where e is the electron charge, n_{j0} is the number concentration of ions in the bulk solution, z_e is the ion valence, k_b is the Boltzmann constant, and T_k is the absolute temperature.

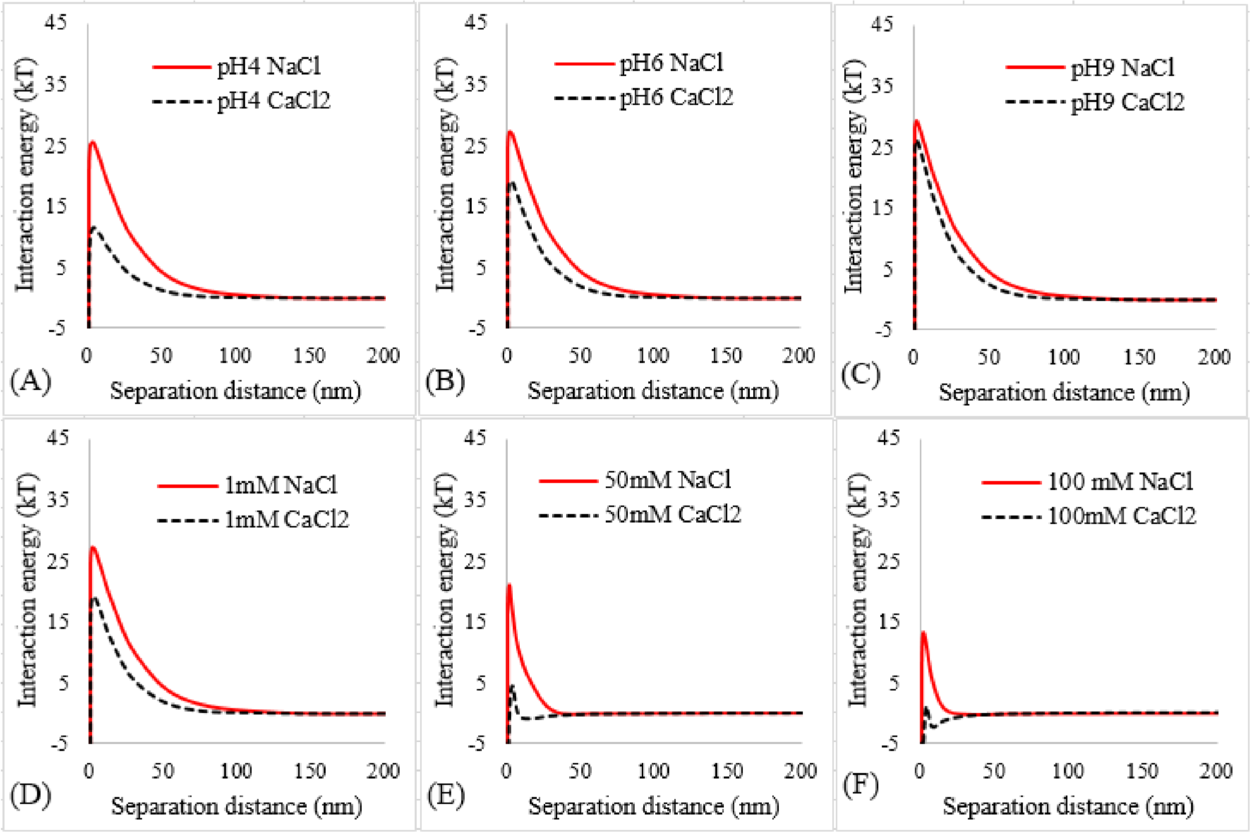

Fig. S1 DLVO energy between N-CDs under different solution conditions.

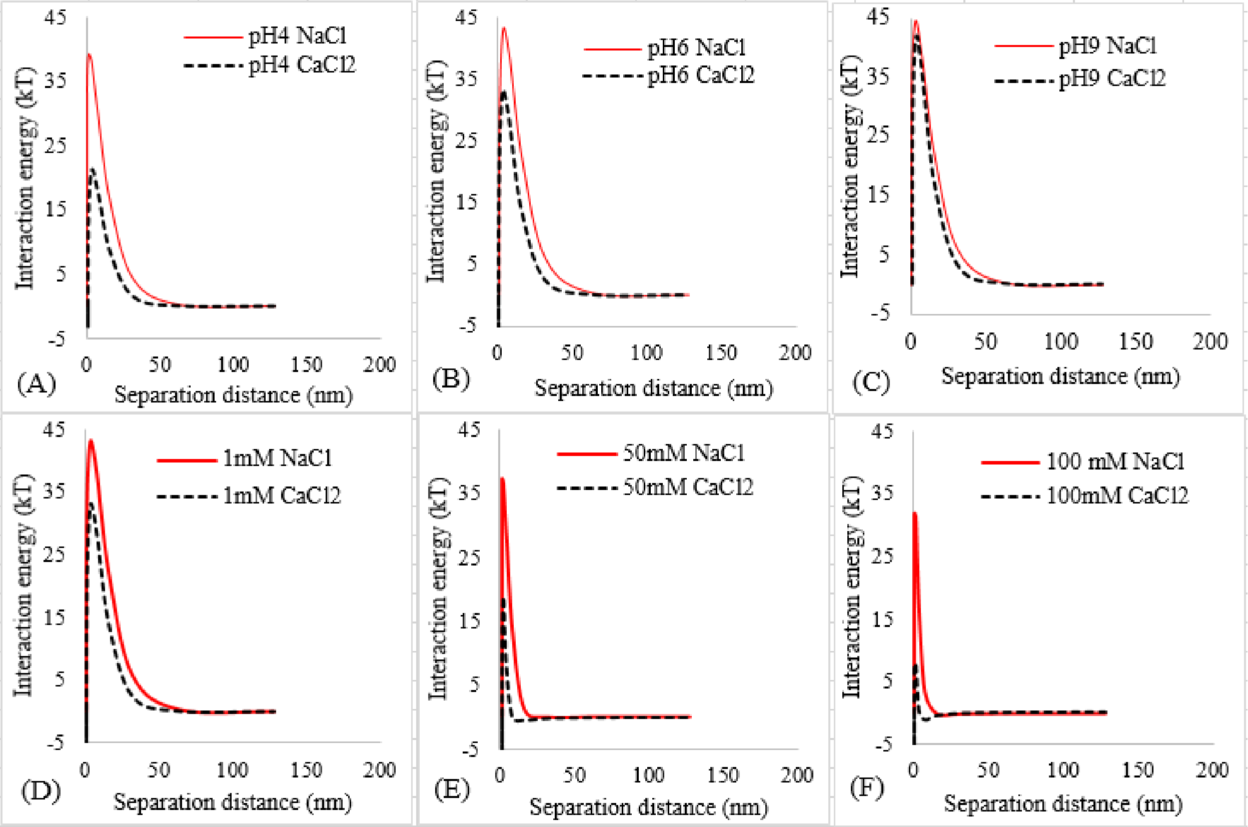

Fig. S2 DLVO energy between N-CDs and quartz surfaces under different solution conditions.

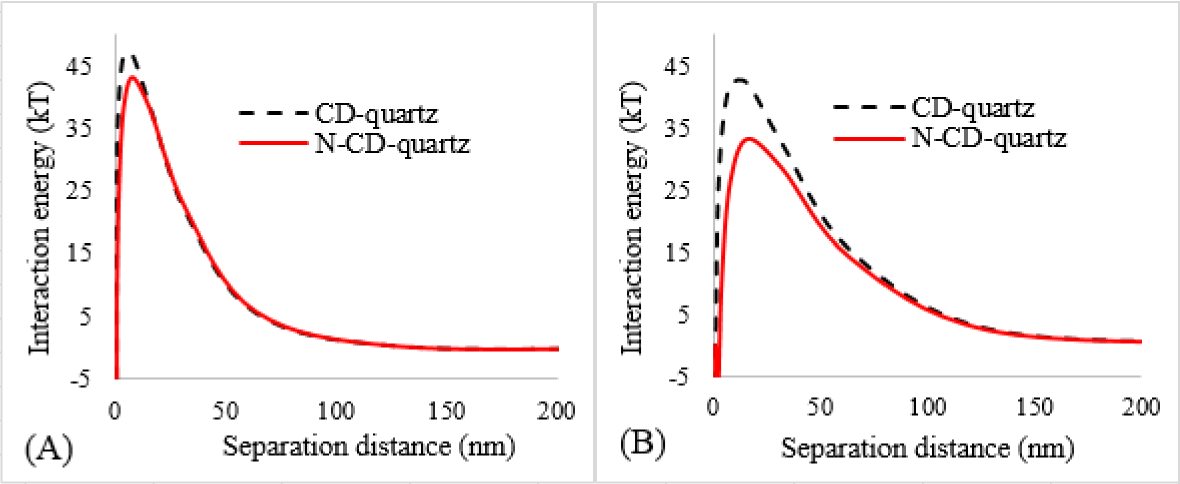

Fig. S3 DLVO energy between N-CDs/CDs and quartz surfaces (pH 6). A) In 1mM NaCl solution and B) in 1mM $CaCl_2$ solution.

Table S1: The maximum energy barrier (Φmax) and Secondary minimum (Φmin2) calculated by DLVO theory.

pH	IS (mM NaCl)	IS (mM $CaCl_2$)	N-CDs zeta potential-mV (±2.5)	quartz grains zeta potential-mV (±2.5)	N-CDs hydrodynamic diameter (±5)	N-CD and N-CD		N-CD and Quartz	
						Φ_{max}[a] (KT)	Φ_{min2}[b] (KT)	Φ_{max}[a] (KT)	Φ_{min2}[b] (KT)
4	1	0	-21.6	-40	44.3	25.5	-0.0006	39.2	-0.0003
6	1	0	-26.2	-65	30.7	27.1	e	43.3	e
9	1	0	-33.5	-80	19.4	29.3	e	44.7	e
6	50	0	-21.5	-51.6	67.1	20.9	-0.05	37.2	-0.05
6	100	0	-15.8	-44.6	75.2	13.2	-0.17	31.7	-0.08
4	0	1	-14.5	-37.5	51.1	11.5	-0.001	21.3	-0.001
9	0	1	-28.2	-75.3	25.5	26.1	-0.0002	41.9	-0.0002
6	0	1	-21	-61.2	36.3	19.1	-0.0006	33.1	-0.0005
6	0	50	-10.2	-45.1	121.5	4.5	-0.93	18.9	-0.41
6	0	100	-8.6	-37.2	136.6	0.9	-2.3	7.8	-1.2

a: The maximum energy barrier (Φmax) calculated by DLVO theory

b: The secondary minimum (Φmin2) calculated by DLVO theory.

The maximum energy barrier (Φmax) between N-CDs-quartz surface (Fig S1) and N-CDs (Fig S2) decreased with increase in IS.

The calculated DLVO energy profiles indicate low repulsive energy barrier between N-CDs and N-CDs-quartz surface in $CaCl_2$ solutions compare to NaCl solutions (Table S1).

The secondary energy minimums get deeper with the increasing IS from 1 to 100 mM for both NaCl and $CaCl_2$ solutions. Furthermore, the separation distance for secondary minimum attractive region decreased with the increasing IS (Fig S2 and S1).

The primary energy barrier between N-CDs-quartz surface and N-CDs increases with the increase in pH. Secondary minimum position between N-CDs-quartz surface (Fig S1) and N-CDs (Fig S2) is nearly constant at different pHs.

.

References

1) Bradford S A, Torkzaban S (2008) Colloid transport and retention in unsaturated porous media: a review of interface-, collector-, and pore-scale processes and models. Vadose Zone J 7(2): 667-681.
2) Bradford S A, Torkzaban S, Walker S L (2007) Coupling of physical and chemical mechanisms of colloid straining in saturated porous media. Water Res 41 (13): 3012–3024.
3) Fan W, Jiang X H, Yang W, Geng Z, Huo M X, Liu Z M, Zhou H (20150 Transport of graphene oxide in saturated porous media: Effect of cation composition in mixed Na–Ca electrolyte systems. STOTEN 511: 509 515.
4) Feriancikova L, Xu S P (2012) Deposition and remobilization of graphene oxide within saturated sand packs. J Hazard Mater 235: 194.
5) Gregory J (1981) Approximate expressions for retarded van der waals interaction. Journal of Colloid and Interface Science 83(1): 138-145.
6) Jiang X, Tong M, Lu R, Kim H (2012) Transport and deposition of ZnO nanoparticles in saturated porous media. Colloids and Surfaces A: Physicochemical and Engineering Aspects 401: 29–37.
7) Lanphere J D, Luth C J, Walker S L (2013) Effects of Solution Chemistry on the Transport of Graphene Oxide in Saturated Porous Media. Environmental Science & Technology 47(9): 4255–4261.
8) Trefalt G, Montes Ruiz-Cabello F J, Borkovec M. (2014) Interaction Forces, Heteroaggregation, and Deposition Involving Charged Colloidal Particles. J Phys Chem B 118: 6346-6355.
